# Morphologic and Molecular Characterization of a Strain of Zika Virus Imported into Guangdong, China

**DOI:** 10.1371/journal.pone.0169256

**Published:** 2017-01-17

**Authors:** Shufen Li, Yongxia Shi, Kui Zheng, Jun Dai, Xiaobo Li, Shuai Yuan, Ling Chen, Jicheng Huang

**Affiliations:** 1 Inspection and Quarantine Technology Center, Guangdong Entry-Exit Inspection and Quarantine Bureau, Guangzhou, P. R. China; 2 State Key Laboratory of Respiratory Disease, The First Affiliated Hospital of Guangzhou Medical University, Guangzhou, P. R. China; University of California Davis, UNITED STATES

## Abstract

The recent outbreaks of Zika virus (ZIKV) disease have caused worldwide concerns. Guangdong province is one of the commercial centers in China and communicates frequently with the epidemic areas. To date, 65.2% of the ZIKV infection cases in China were imported via port of entry in Guangdong. The continuous surveillance of imported cases is crucial for the prevention and control of potential ZIKV infection outbreak in China. In this study, a strain of ZIKV was isolated from the serum of a 6-year-old child returning from Venezuela. The morphology of the ZIKV was analyzed *in vivo* and *in vitro* by electron microscopy, and clusters of virus particles were found in the loose cytoplasmic membrane structures. The genomic sequence of the isolated ZIKV was determined, and the alignment and phylogenetic analysis identified one unique amino acid substitution occurring in the non-structural protein 4B (NS4B), and the isolated virus belonged to the Asian lineage.

## Introduction

Zika virus (ZIKV) is an emerging arbovirus, which belongs to the *Flavivirus* genus. Like other flaviviruses, ZIKV is an enveloped virus with a positive single-stranded RNA genome, which contains one open reading frame encoding a polyprotein that is cleaved into three structural proteins (including capsid protein, premembrane/membrane protein and envelope protein) and seven nonstructural proteins (NS1, NS2A, NS2B, NS3, NS4A, NS4B and NS5) [1]. ZIKV is mainly transmitted by *Aedes* species mosquitoes [2,3]. Recent studies indicated that ZIKV could be detected in urine, saliva and semen [4–6] other than serum, and sexual transmission of ZIKV have been reported [7,8]. In addition, infective ZIKV was isolated from urine and saliva of acute phase patients, suggesting that ZIKV might be transmitted through urine and saliva [9].

ZIKV was first isolated from a sentinel rhesus monkey in Uganda in 1947 [10], with periodic human infections in Africa and South Asia [2,11,12]. The first large outbreak of ZIKV occurred in 2007 in Micronesia [13], ZIKV was then identified in French Polynesia, New Caledonia, Cook Islands and Easter Island of Chile during 2013–2014 [14–18]. To date, ZIKV disease has rapidly spread to more than 60 countries and regions ranging from Africa, Americas, and the Pacific to Europe and Asia, since the initial outbreak in South Americas in early 2015. In most circumstance, ZIKV infection causes self-limited diseases including fever, rash, conjunctivitis and joint pain. However, the rapid transmission and the direct link between ZIKV infection and fetal microcephaly [19–22] has made this virus a public health emergency of international concern.

According to the National Health and Family Planning Commission of China, 23 cases of ZIKV infection imported into Jiangxi, Guangdong, Zhejiang and Beijing have been identified since February 2016. Guangdong, a large province in South China, shares frequent communications with the epidemic areas. In the Enping city of Guangdong, 180,000 people are living in Venezuela, where the large outbreak of ZIKV infection occurs. To date, 65.2% (15/23) of the ZIKV infection cases in China were imported via Guangdong port, most of which were back from Venezuela (Table 1). Furthermore, wide distribution of *Aedes* species mosquitoes in Guangdong raises the risk of epidemic. Thus, the continuous surveillance of imported ZIKV cases is crucial for the prevention and control of further ZIKV outbreak in China. In this study, we identified and isolated an imported ZIKV strain from the serum of an infected child returning from Venezuela, and the morphologic and molecular characterization was conducted.

**Table 1:** Information of ZIKV infection cases imported into Guangdong*.

No.	Age	Gender	Date of diagnosis	Country origin	Symptom
1	34	Male	Feb. 8, 2016	Venezuela	Headache, fever
2	28	Male	Feb. 12, 2016	Venezuela	Fever, rash
3	38	Male	Feb. 19, 2016	Samoa	Fever, chills
4	38	Male	Feb. 23, 2016	Samoa	Fever
5	8	Male	Feb. 23, 2016	Samoa	Fever
6^#^	6	Male	Feb. 25, 2016	Venezuela	Fever
7	23	Male	Feb. 26, 2016	Venezuela	Rash
8^#^	8	Female	Feb. 27, 2016	Venezuela	Rash
9^#^	40	Male	Feb. 29, 2016	Venezuela	Rash
10	19	Male	Mar. 9, 2016	Venezuela	Rash
11	12	Female	Mar. 24, 2016	Venezuela	Rash
12	13	Female	Mar. 29, 2016	Venezuela	Rash
13	7	Female	Apr. 7, 2016	Venezuela	Rash
14	37	Male	May. 14, 2016	Venezuela	Fever, rash
15	32	Female	Jun. 5, 2016	Venezuela	Rash

* This information was obtained from the website of Guangdong Health and Family Planning Commission (http://www.gdwst.gov.cn/contentsearch/default.jsp) and National Health and Family Planning Commission of China (http://www.nhfpc.gov.cn/).

^#^ The family clustering case. 6. The child enrolled in this study, 8. The sister of this child, 9. The father of this child.

## Materials and Methods

### Specimen

Serum, urine and saliva samples were collected from a 6-year-old child, returning from Venezuela. The initial symptom of the patient was fever (38°C). And the samples of patient were sent to the health and quarantine lab with a written consent from patient’s guardian, in order to confirm the infection of ZIKV and to conduct the subsequent study.

### Viral RNA extraction and detection

Viral RNA was extracted from 140 μl of serum, urine and saliva samples, respectively, by using QIAamp Viral RNA kit (QIAGEN, Germany) according to manufacturer’s instruction. ZIKV RNA was determined by using real time RT-PCR on an ABI 7500 Fast Instrument. The primer set used to detect viral RNA was: forward primer, 5’-GTGACGCCACCATGAGCTATGA-3’; reverse primer, 5’- TGATGGCAGGTTCCGTACACAAC-3’; and probe, 5’- /FAM/CCAAGTTGACGTCGTGTTGCACCAGCA/BHQ1/3’.

### Determination of viral load

The ZIKV RNA in serum from patient and in tissues from ZIKV-infected mice was extracted and measured by one-step quantitative reverse transcriptase PCR (qRT-PCR), comparing with a standard curve produced by using a serial of 10-fold diluted ZIKV RNA (obtained from *in vitro* transcription). The primer set used to determine viral load was: forward primer, 5’-TGGAGGTTGTGTCACCGT-3’; reverse primer, 5’-CAGTAGGATCTTACCTCC-3’; and probe, 5’- /FAM/GGCACAGGACAAACCGACTGTCGACA/BHQ1/3’.

### Virus isolation

One-day-old Kunming suckling mice (n = 10) were inoculated intracranially with pretreated acute phase serum (Ct value = 25, viral load 5.34±0.16×10^7^ copies/ml) from the patient, and were bred together with the mother mouse under specific-pathogen-free condition (temperature: 23±2°C, relative humidity: 40–70%). The mother mouse received enough food and water, and the suckling mice were breast fed by the mother. The physical condition of inoculated mice was monitored twice daily. We had a protocol in place for the early euthanasia/humane endpoints but that none of the mice became severely ill or moribund during the experiment. All of the mice were euthanized by carbon dioxide inhalation 10 days post-inoculation, and the organs including brain, kidney, lung and heart were collected and homogenized in DMEM medium (Gibco, USA) supplemented with 2% inactivated fetal bovine serum (Gibco, USA). The homogenized supernatant of brain (*Ct* value = 18) was used to inoculate BHK-21 and Vero cells (BHK-21 and Vero cell lines were provided by Professor Ling Chen on Mar 15, 2016) to further amplify the virus.

### Electron microscopy (EM)

For tissue observation, a small piece of brain tissue was immediately fixed with 2.5% (W/V) glutaraldehyde in 0.1 M sodium chloride followed by post-fixation in 1% osmium tetroxide in 0.1 M cacodylate buffer. Tissue was then dehydrated in a serial of increasing concentrations of ethanol. The sample was then embedded in epoxy resin. Ultrathin sections were prepared using a Leica UltraCut S Microtome and stained with uranyl acetate and lead citrate. For cell observation, infected Vero cells were fixed in 2.5% (W/V) glutaraldehyde in 0.1 M sodium chloride at 72 h post infection, and processed for EM analysis as described above. All of the EM samples were examined by a JEM-1400 transmission electron microscope (JEOL, Japan).

### RT-PCR and genome sequencing

Eight pairs of primers listed in Table 2 were used to amplify and sequence the entire genome of this ZKIV strain. The amplification was conducted with QIAGEN OneStep RT-PCR Kit (QIAGEN, Germany) followed by the steps of 50°C for 30 min, 95°C for 15 min, and 30 cycles of 94°C for 30 sec, 55°C for 30 sec, 72°C for 1 min, and a final extension at 72°C for 5 min. The PCR products were purified and submitted for sequencing.

**Table 2:** List of primers used to obtain the entire coding sequence (CDS) of ZIKV.

Primer set No.	Amplification location (nt)	Forward primer (5’-3’)	Reverse primer(5’-3’)
1	-26-1524	GGATTTGGAAACGAGAGTTTC	GAACCACTCCTTGTGAACCA
2	1427–2903	TAGGACTTGATTGTGAACCGA	TCTTCTCTAACCTTGAGCCA
3	2782–4298	GTCGTGGATGGTGACACAC	CATGTGATGTCACCTGCTCT
4	4088–5619	TGCTGTTGCTCACAAGGAGT	CTCATTGCCGTTCCTCACG
5	5500–7110	ATGGACACCGAAGTGGAAGT	GTAGCAACCTATCATTAGCA
6	6931–8410	ATCTATGCTGCCTTGACAACT	TTTCAATGCGGTTACCAATGAT
7	8266–10270	ACGAGCCAGCTCCTCTTG	ACAGCACTCCAGGTGTAGAC
8	9480–10313	GAGGTCAGAGAAAGTGACC	CTGTGGCTGACTAGCAGG

### Alignment and phylogenetic analysis

The entire coding sequence (CDS) of the isolated ZIKV was acquired and compared to worldwide reference sequences of different strains available in GenBank. The nucleotide sequences were aligned, edited, and phylogenetically analyzed by using MEGA software version 5.2. Trees were constructed by Neighbor-joining method, with bootstrap value obtained from 1000 replicates.

### Statistical analysis

A two-tailed Student’s *t* test was used to assume unequal variances for statistical comparisons, and a *P* value of < 0.05 was considered to be statistically significant.

## Results

### The isolation and amplification of Zika virus

**Fig 1:**
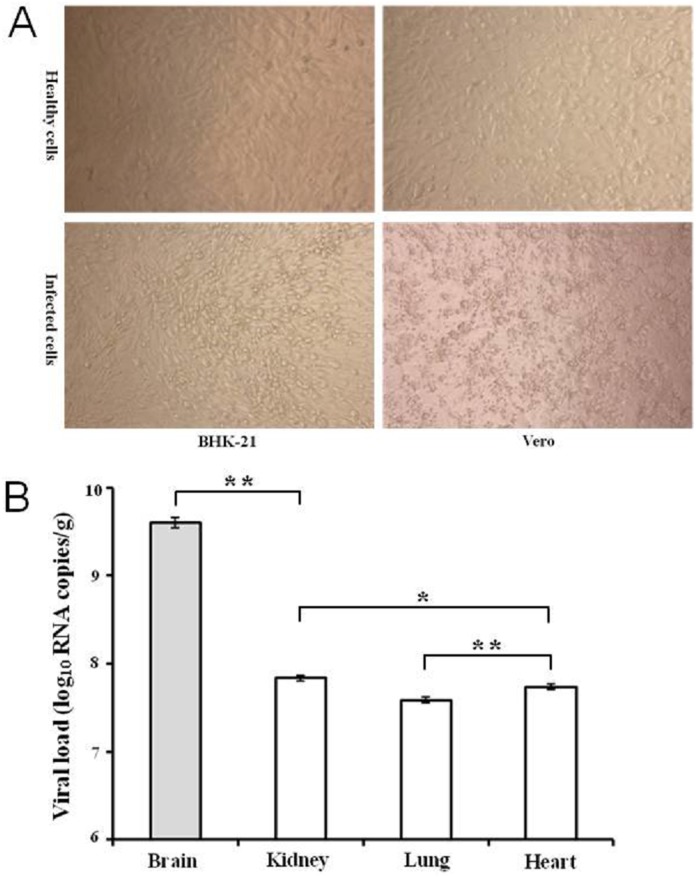
The isolation and amplification of the ZIKV GZ02 strain. (A) The amplification of ZIKV GZ02 strain in BHK-21 and Vero cells infected with the homogenized supernatant of brain. Typical cytopathic effects were observed at 5 days post-infection. (B) The viral loads in tissue samples from ZIKV-infected mice were quantified by qRT-PCR and expressed on a log_10_ scale as viral RNA copies per gram after comparison with a standard curve. The experiments were repeated twice. Error bars represent standard deviation. Data were analyzed by a two-tails student t test. * and ** represent *P* < 0.05 or 0.01, respectively.

The acute phase serum from the infected patient was intracranially inoculated to one-day-old Kunming suckling mice (n = 10). Ten days post inoculation, three of the infected mice showed mild symptoms including emaciation, hypoergia and hind-leg paralysis. Seven of the inoculated mice experienced asymptomatic infection. All of the mice were dissected, and several organs were collected and grinded. Total RNAs of homogenized tissues were extracted and detected by quantitative RT-PCR. ZIKV RNA was detected from the brain tissues of all the infected mice. The supernatant of homogenized brain was used to inoculate BHK-21 and Vero cells to further amplify the virus. Typical cytopathic effects, such as turning round, shrinkage and rupture were observed in both cell lines. Of note, the cytopathic effect in BHK-21 cells was much milder than that in Vero cells (Fig 1A). The ZIKV strain isolated from serum of patient was named GZ02. Comparative detection of the viral loads in various tissues revealed that the viral load in brains was significantly higher than those in kidneys, lungs and hearts (*P* < 0.01); the viral RNA level in hearts was lower than kidney (*P* < 0.05); and the lungs showed the lowest viral burden, that was significantly lower than the virus titers in other organs (*P* < 0.01) (Fig 1B). The results suggested that ZIKV possesses polytropism towards tissues.

### The morphology of ZIKV *in vivo* and *in vitro*

**Fig 2:**
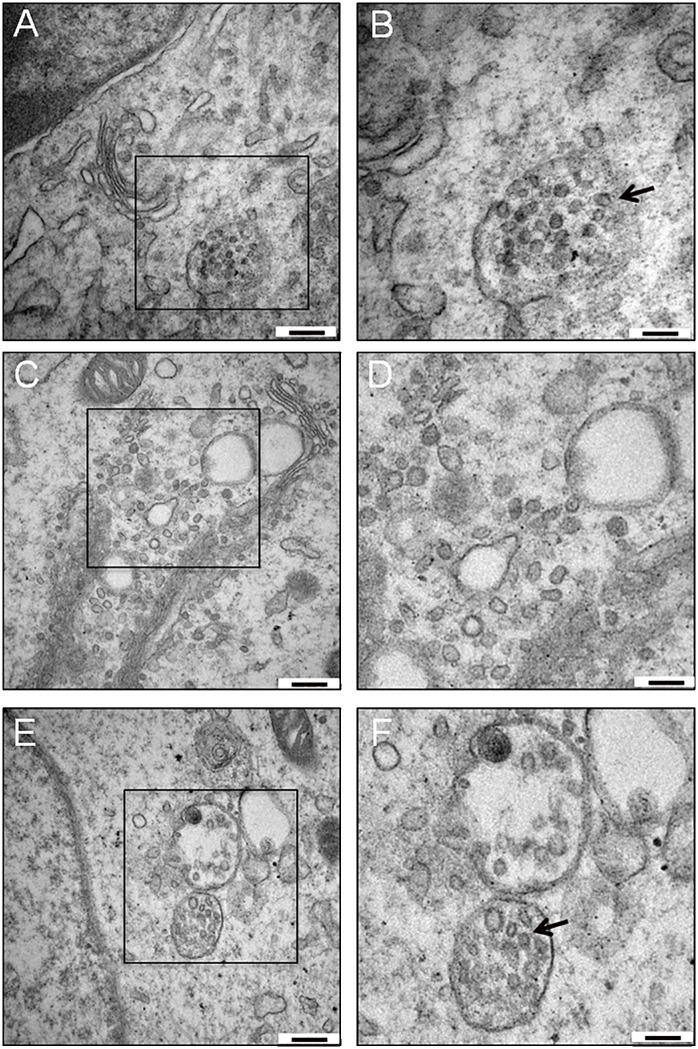
Electron microscopy of ZIKV-infected brain tissue and Vero cells. (A) Ultrathin section image of ZIKV-infected brain tissue. Spherical ZIKV particles were observed in cytoplasmic compartments. (B) Enlarged view of the boxed area in A. Black arrow indicates the presence of ZIKV capsids. (C) Ultrathin section image of infected Vero cells shows a group of virus-induced membrane structures with bright interior, indicating viral replication. (D) Enlargement of the boxed area in (C). (E) Spherical capsids were found in the cytoplasm of infected Vero cells. (F) Enlarged view of the boxed area in E. Black arrow indicates the presence of capsids. Bars represent 200 nm (A, C and E) and 100 nm (B, D and F), respectively.

The morphologies of ZIKV in mouse brain and in Vero cells were further observed by electron microscopy. The electron microscopy of the brain tissue revealed that clusters of ZIKV particles in diameter of approximately 50 nm were observed in cytoplasmic compartments (Fig 2A and 2B). In ZIKV infected Vero cells, the virus-induced intracellular membrane structures with a bright interior were captured in cytoplasm, indicating the replication of ZIKV (Fig 2C and 2D). The presence of clustered spherical particles in loose membrane vesicles was found in the cytoplasm of ZIKV-infected Vero cells (Fig 2E and 2F).

### Alignment and phylogenetic analysis

**Fig 3:**
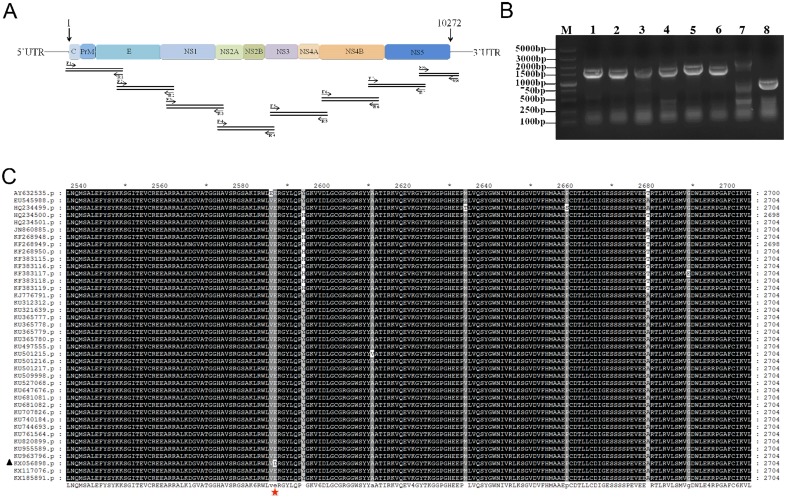
Sequencing strategy and alignment analysis of ZIKV genome. (A) Structure and sequencing strategy of ZIKV genome. One open reading frame encodes a polyprotein, including capsid protein (C), premembrane/membrane protein (PrM), envelope protein (E) and seven nonstructural proteins (NS1, NS2A, NS2B, NS3, NS4A, NS4B and NS5). Eight pairs of primers were designed to amplify the entire CDS of ZIKV. (B) The amplification of the CDS of GZ02 strain using 8 pairs of primers. Eight amplified fragments were visualized by electrophoresis. (C) Alignment analysis of the amino acid sequences. The GZ02 strain is designated in solid triangle, the unique amino acid change was marked with red pentagram.

**Fig 4:**
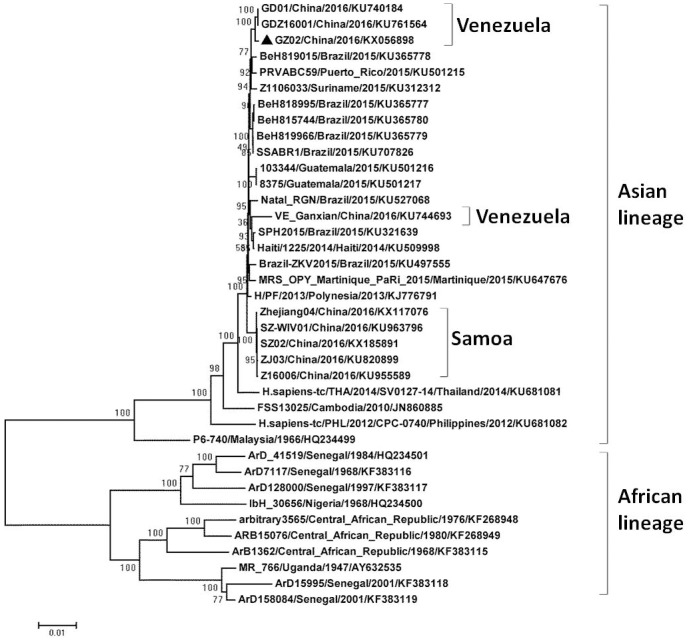
Phylogenetic analysis of ZIKV. The phylogenetic tree was constructed based on the entire CDS of ZIKV by using the neighbor-joining method with bootstrap 1000 replications. The sequences were named according to strain/country/year/GenBank accession number. The GZ02 strain is designated in solid triangle.

In order to obtain the full genome of the isolated ZIKV (named GZ02), eight pairs of specific primers (Table 2) were designed to amplify the overlapping fragments covering the entire CDS (Fig 3A). The correct PCR products with an average length of 1500 base pairs were detected (Fig 3B) and subjected for sequencing and assembly. The complete CDS of isolated strain contains a single long open reading frame (10,272 nt), encoding a polyprotein with 3,423 amino acids. The sequence was submitted to GenBank under accession no. KX056898. Alignment of the amino acid sequences of our ZIKV isolate with other 39 strains available in GenBank revealed that a unique amino acid substitution (E2587D) occurred in NS4B (Fig 3C), which may represent an occasional event or a consequence of an adaptation of the virus to host. Phylogenetic analysis of the new isolated ZIKV and other available strains indicated that all of ZIKV strains were clustered into Asian and African lineages, and the isolated strain belongs to the Asian lineage (Fig 4).

## Discussion

The pandemic of ZIKV infection occurred in South America, West Africa and Caribbean since last year, and rapidly spread to other countries and regions. First imported case into China was identified in February 2016, followed by another 22 more cases. On 25 February, the serum, urine and saliva samples of a six-year old boy returning from Venezuela were identified to be nucleic acid positive for ZIKV. The other three members in his family were monitored for infection, subsequently. On 27 February, the boy’s sister had a rash and was diagnosed of ZIKV infection, and both of the parents were detected positive for ZIKV RNA without symptom. On 28 and 29 February, the father developed a rash and was clinical confirmed as an infected case. The mother showed no symptom throughout the entire observation and was defined as asymptomatic infection. More investigations are needed to elucidate the family clustering infection.

Acute phase serum of the patient was used to intracranially inoculate Kunming suckling mice. Three of the ten infected mice developed symptoms of emaciation, hypoergia and hind-leg paralysis. The rest of the mice experienced asymptomatic infection. All of the infected mice survived, which indicated that ZIKV was not lethal to outbred Kunming suckling mouse. The detection of virus in different organs suggested that ZIKV is polytropic to multiple tissues with highest virus load in brains, lower in kidneys and hearts, and minimum in lungs (Fig 1B).

The morphology of ZIKV *in vivo* and *in vitro* was analyzed by electron microscopy (Fig 2). The virus-induced intracellular membrane vehicles are closely associated with the replication of flaviviruses. Like other flaviviruses, the replication of ZIKV occurs in the cellular cytoplasm [23,24]. The intracellular membrane structures with a bright interior were observed in cytoplasm. Predicted ZIKV particles were captured in loose membrane compartments. The observation of ZIKV-infected cells suggested a morphologic pattern consistent with other flaviviruses [25].

The entire CDS of the isolated strain was sequenced. Alignment of the amino acid sequences indicated that a unique amino acid substitution occurred in NS4B (Fig 3C). NS4B is one of the essential components of the endoplasmic reticulum (ER) membrane-associated replication complex, which is indispensable for the replication of *flavivirus* [26,27], thus further studies are needed to clarify the biological significance of this mutation. The ZIKV infection cases in China were imported either from Venezuela or Samoa [28,29], which formed three separated clusters in the Asian lineage (Fig 4). The result is consistent with previous works showing that the recent outbreaks of ZIKV derived from the Asian lineage [30]. The worldwide transmission and the association of ZIKV with the fetus microcephaly and Guillain-Barré syndrome should be of great concern to researchers, more works are needed to be carried out to prevent and control the further outbreaks of ZIKV infection.

## Compliance with Ethical Standards

No conflict exists: Author Shufen Li declares that she has no conflict of interest. Author Yongxia Shi declares that she has no conflict of interest. Author Kui Zheng declares that he has no conflict of interest. Author Jun Dai declares that he has no conflict of interest. Author Xiaobo Li declares that he has no conflict of interest. Author Shuai Yuan declares that she has no conflict of interest. Author Ling Chen declares that he has no conflict of interest. Author Jicheng Huang declares that he has no conflict of interest.

Ethical approval: The study associated with human samples has been approved by the Ethics Committee of Guangdong Inspection and Quarantine Technology Center. The information and samples of patients were collected under approval. A written consent from the guardian on behalf of the child was obtained. The animal experiment was approved by the Experimental Animal Ethics Committee of Guangdong Inspection and Quarantine Technology Center. The Kunming suckling mice were maintained together with the mother mouse under specific-pathogen-free condition. All the mice were monitored twice daily and euthanized at the experimental endpoint.

Consent for publication: Consent to publish has been obtained from all subjects to report individual patient data.

## Supporting Information

S1 Dataset The viral load in patient’s serum. Total RNAs in patient’s serum were extracted and detected by quantitative RT-PCR. The viral load in 1ml serum was calculated.
(XLSX)

S2 Dataset The viral load in tissue samples. The ZIKV RNA in tissue samples from ZIKV-infected mice was extracted and measured by qRT-PCR. The data was expressed as viral RNA copies per gram.
(XLSX)

S3 Dataset The original ultrathin section images of ZIKV-infected brain tissue and Vero cells. Figure A in S3 dataset. The original ultrathin section images of ZIKV-infected brain tissue in Fig 2A and 2B. Figure B in S3 dataset. The original ultrathin section images of ZIKV-infected Vero cells in Fig 2C and 2D. Figure C in S3 dataset. The original ultrathin section images of ZIKV-infected Vero cells in Fig 2E and 2F. Bars represent 500 nm.
(RAR)
